# Newly recorded psychiatric diagnoses among 21 million youth before, during, and after COVID‐19 restrictions: A nationwide interrupted time‐series analysis

**DOI:** 10.1111/pcn.70117

**Published:** 2026-08-10

**Authors:** Naomi Matsumoto, Hisashi Ishida, Shunsaku Hayase, Takahiro Asada, Yoshie Shigeyasu, Ayumi Okada, Tomoya Hirota, Takashi Yorifuji

**Affiliations:** ^1^ Department of Epidemiology, Faculty of Medicine, Dentistry and Pharmaceutical Sciences Okayama University Okayama Japan; ^2^ Department of Pediatrics Okayama University Hospital Okayama Japan; ^3^ Department of Epidemiology Okayama University Graduate School of Medicine, Dentistry and Pharmaceutical Sciences Okayama Japan; ^4^ Department of Neuropsychiatry Okayama University Hospital Okayama Japan; ^5^ Department of Pediatrics Okayama University Graduate School of Medicine, Dentistry and Pharmaceutical Sciences Okayama Japan; ^6^ Department of Child Development and Mental Health Okayama University Graduate School of Medicine, Dentistry and Pharmaceutical Sciences Okayama Japan

**Keywords:** adolescent, attention deficit disorder with hyperactivity, COVID‐19, interrupted time series analysis, mental disorders

## Abstract

**Aim:**

The COVID‐19 pandemic had substantial effects on youth mental health. Most previous studies focused on the initial pandemic years, leaving the post‐restriction period underexplored. We examined trajectories of newly recorded psychiatric diagnoses among Japanese youths across two policy shifts: March 2020 school closures and May 2023 reclassification of COVID‐19 as an influenza‐equivalent Category V disease.

**Methods:**

We conducted an interrupted time‐series analysis using the National Database of Health Insurance Claims, covering over 99% of the Japanese population. The study included 21 million individuals aged under 19 years to analyze monthly trends in first recorded psychiatric diagnoses from January 2018 to March 2024. Segmented negative binomial regression was used to estimate shifts in levels and trends following both policy interventions.

**Results:**

Following the 2020 closures, eating disorders showed sex‐specific patterns: males showed an immediate 28.8% decrease (IRR 0.712, 95% CI 0.619–0.819), whereas females showed a 28.4% increase (IRR 1.284, 95% CI 1.045–1.576). Newly recorded ADHD diagnoses showed an 8.1% immediate decrease (IRR 0.919, 95% CI 0.847–0.997). During the restriction period, anxiety/stress‐related and mood disorders showed apparent surge patterns following infection waves. After restrictions were lifted in 2023, eating disorders among males showed an upward level shift (IRR 1.214, 95% CI 1.076–1.370). Neurodevelopmental disorders showed preliminary downward trends in the post‐restriction period.

**Conclusion:**

This nationwide study of 21 million youths suggested differential diagnostic patterns across pandemic phases. These contrasting trajectories highlight the need for subgroup‐level post‐pandemic diagnostic surveillance.

The COVID‐19 pandemic profoundly affected pediatric and adolescent mental health worldwide.^1^, ^2^ Accumulating evidence suggests that the pandemic's influence varied considerably across psychiatric diagnostic categories. Previous studies have indicated that depression, anxiety, and eating disorders showed an immediate and sharp increase following the pandemic onset, and that this increase may have been driven by acute psychological distress and social isolation —as evidenced by rising prevalence of clinically elevated depressive and anxiety symptoms^1^ and increased diagnostic incidence of eating disorders.^3^, ^4^ In contrast, neurodevelopmental disorders, including attention‐deficit/hyperactivity disorder (ADHD) and autism spectrum disorder (ASD), exhibited a more complex trajectory. Whereas some children with these conditions experienced a temporary mitigation of symptoms during school closures owing to the removal of academic and social stressors,^5^, ^6^ many experienced a later exacerbation and a subsequent rise in clinical presentations as restrictions were lifted and structured schooling resumed, suggesting a possible role of person–environment mismatch.^7^, ^8^

Despite these findings, most longitudinal research has focused on the first few years of the pandemic. Although recent studies have begun to examine post‐restriction trajectories, these have typically been limited to single diagnostic categories — for example, neurodevelopmental disorders in young children^9^ or ADHD diagnoses among individuals younger than 30 years^10^ in Canada — leaving open the question of how multiple psychiatric disorders evolve simultaneously, and how their trajectories vary by sex and age within a single population. Our previous interrupted time‐series (ITS) analysis using a multicenter electronic health records database demonstrated that initial pandemic effects on newly recorded cases of pediatric psychiatric disorders varied by disorder type, sex, and age, with more pronounced effects in females and adolescents.^11^ However, it remains unclear whether the observed increase in psychiatric diagnoses represents a transient adjustment reaction or a sustained shift in clinical presentation, characterized by a persistently elevated number of newly recorded cases. Determining whether the pandemic has led to lasting changes in the developmental trajectories of youths is an important public health priority.

The Japanese context provides a unique opportunity to examine these longitudinal trends. Unlike many other countries, Japan did not implement mandatory lockdowns but instead maintained long‐term, stringent social distancing measures through strong social conformity pressure,^12^ such as “silent lunches” (*moku‐shoku*)—a practice in which all students were required to eat facing forward in complete silence without any conversation or face‐to‐face interaction—and the cancellation of school events. This *moku‐shoku* policy, which persisted for over 3 years in many schools, fundamentally altered the social dynamics of school life, as lunch periods are traditionally key opportunities for peer interaction and social development in Japanese schools.^13^ The reclassification of COVID‐19 as a Category V infectious disease (the lowest‐risk category under Japan's Infectious Diseases Control Law, equivalent to seasonal influenza) in May 2023 marked a distinct transition from these protracted restrictions to an open social environment. Furthermore, Japan's universal health insurance system permits comprehensive observation of psychiatric trends across the entire population through the National Database of Health Insurance Claims of Japan (NDB).^14^

The study objective was to elucidate the long‐term, longitudinal trajectories of new diagnoses of psychiatric conditions among children and adolescents. We focused on the differential effects of the pandemic on two distinct disease clusters: (i) mood disorders and anxiety/stress‐related disorders, hypothesized as showing immediate reactions to pandemic‐related distress, and (ii) neurodevelopmental disorders, hypothesized as manifesting through shifts in person–environment fit during social transitions, given evidence that neurodevelopmental symptoms are substantially modulated by environmental demands.^15^ Eating disorders were examined separately, as they may share features of both acute stress responses and person–environment fit dynamics. By analyzing data surrounding two pivotal points—the initial nationwide school closures in 2020 and the transition to a Category V disease in 2023—this study aimed to characterize the long‐term trajectories of new diagnoses for pediatric psychiatric disorders and to examine how these trajectories differed across diagnostic categories, sex, and age.

## Methods

### Study design and setting

We conducted a nationwide ITS analysis using segmented negative binomial regression to evaluate the level and trend of new psychiatric diagnoses among children and adolescents.^16^, ^17^

We examined two policy shifts: (i) March 2020, marked by nationwide school closures and the initiation of stringent social distancing protocols, including *moku‐shoku*,^13^ and (ii) May 2023, when COVID‐19 was reclassified as Category V, and the restrictions were formally terminated.^18^, ^19^ The study period spanned 75 months (January 2018–March 2024), divided into three phases: pre‐pandemic (26 months), restriction (38 months), and post‐restriction (11 months). The post‐restriction period (11 months) was determined by data availability (through March 2024).

### Data source

Patient data were retrieved from the NDB. Maintained by the Ministry of Health, Labour and Welfare, the NDB is an administrative database covering approximately 99% of all healthcare insurance claims under Japan's universal health insurance system,^14^ including claims fully funded by public funds such as medical assistance for public assistance recipients. It includes longitudinal records of inpatient and outpatient visits for nearly 126 million citizens from birth until death. The NDB's population‐based coverage minimizes selection bias and captures psychiatric consultations across all regions of Japan. Data were retrieved in March 2025, which ensured that the analysis included the most recent records up to the end of the 2023 Japanese fiscal year (March 31, 2024) (see Methods S1).

### Participants and Case Identification

The study population included the entire pediatric population in Japan, totaling approximately 20.97 million as of March 2020 (0–4 years: 4.65 million; 5–9 years: 5.14 million; 10–14 years: 5.37 million; 15–19 years: 5.81 million).^20^ Sex was classified based on insurance enrollment records, which reflect sex registered at birth in the Japanese civil registration system. Newly recorded cases were identified through a three‐step process. First, we extracted claim records with a target diagnosis between January 2017 and March 2024 and organized them by individual identifier. Second, those with a previous record of the same diagnosis, with a start date between 2008 and 2016, were excluded as prevalent cases. Third, we applied an additional 12‐month washout period by excluding those whose first diagnosis was recorded in 2017. This step was taken to minimize misclassification due to provider transfers or insurer changes, ensuring that ‘newly recorded cases’ specifically refers to individuals whose first recorded diagnosis in the NDB (available from 2008) occurred on or after January 1, 2018 (see Methods S1).

### Diagnostic validity and categorization

Only confirmed diagnoses were included; records flagged as “suspected” were excluded. Coding‐based diagnostic validity may vary by category (e.g., non‐specific codes such as F50.9); see Methods S2. Diagnostic categories, age‐based inclusion criteria, specific ICD‐10 and Japanese claims codes, and comorbidity handling are described in detail in Methods S2 and Table S1.

### Statistical analysis

We performed ITS analysis using segmented negative binomial regression,^16^ with month dummy variables for seasonality and Newey–West HAC standard errors to account for residual autocorrelation (Portmanteau test: all *P* < 0.001).^21^ Monthly population estimates from the Statistics Bureau of Japan were included as an offset term.^20^ Given that analyses were conducted across multiple outcomes and subgroups, both unadjusted *P*‐values and FDR‐adjusted *q*‐values (Benjamini–Hochberg procedure; q < 0.05)^22^ are reported to allow transparent interpretation. The full model specification, variable definitions, and interpretation of incidence rate ratios (IRRs) are provided in Methods S3. All analyses were performed using Stata version 19.0 (StataCorp LLC, College Station, TX, USA).

### Ethics approval

The Institutional Review Board of the Graduate School of Biomedical Sciences, Okayama University (No. 2406–032) approved this study, and it conformed to the provisions of the Declaration of Helsinki. The requirement for informed consent was waived because the study used anonymized administrative claims data with no direct patient contact. Patient anonymity has been preserved.

## Results

Table 1 shows the monthly mean number of newly recorded diagnosis cases across diagnostic categories and study periods. Anxiety/stress‐related disorders (*n* = 1,432,284) showed a relatively balanced sex distribution, with cases distributed across all age groups. Mood disorders (*n* = 459,517) demonstrated female predominance (female‐to‐male ratio approximately 1.5:1), with the highest number of new diagnoses in the 15– <19 years age group. Eating disorders (*n* = 53,075) showed a strong female predominance (approximately 2.4:1) and peaked in adolescence. ADHD (*n* = 612,598) showed male predominance (approximately 2.7:1), with the highest number of new diagnoses in the 5–9 years age group. ASD (*n* = 872,260) also showed male predominance (approximately 2.2:1) but with a more even age distribution. Temporal variations in monthly case numbers were observed across all diagnostic categories, with notable fluctuations during the restriction period (Table 1).

**Table 1:** Monthly mean number of newly recorded diagnosis cases by diagnostic category, sex, and age group across study periods (2018–2024).

Characteristics		Total period	Pre‐pandemic	During restrictions	Post‐restriction
Anxiety/Stress‐Related Disorders (*n* = 1,432,284)					
All		19,097.1 (2619.9)	18,666.5 (1693.5)	19,438.3 (3228.7)	18,936.4 (2024.7)
Sex	Male	8798.6 (1013.5)	8906.4 (712.4)	8744.3 (1223.8)	8731.2 (862.6)
	Female	10,298.6 (1673.6)	9760.1 (1033.7)	10,694.0 (2035.7)	10,205.2 (1168.1)
Age	Age 5–9	4502.2 (648.6)	4692.7 (640.2)	4373.5 (640.3)	4496.6 (643.8)
	Age 10–14	6688.0 (1125.0)	6482.1 (819.0)	6857.7 (1349.4)	6588.5 (849.1)
	Age 15–<19	7906.9 (1180.1)	7491.7 (675.8)	8207.1 (1452.7)	7851.2 (749.3)
Mood disorders (*n* = 459,517)					
All		6126.9 (1360.4)	5111.8 (561.2)	6703.9 (1489.9)	6532.7 (755.1)
Sex	Male	2460.3 (431.8)	2222.6 (224.3)	2582.6 (503.2)	2599.5 (303.2)
	Female	3666.6 (949.5)	2889.2 (347.5)	4121.4 (1002.6)	3933.2 (462.6)
Age	Age 5–9	310.7 (61.8)	310.6 (58.7)	313.1 (67.9)	302.4 (50.2)
	Age 10–14	1708.4 (439.2)	1429.5 (235.6)	1876.1 (484.6)	1788.2 (322.3)
	Age 15–<19	4107.9 (934.1)	3371.7 (360.1)	4514.8 (1008.2)	4442.2 (459.6)
Eating disorders (*n* = 53,075)					
All		707.7 (113.9)	711.8 (76.6)	711.9 (141.5)	683.1 (80.6)
Sex	Male	206.1 (47.2)	247.1 (31.9)	175.7 (36.5)	214.2 (33.0)
	Female	501.6 (95.5)	464.7 (53.0)	536.3 (114.7)	468.9 (50.9)
Age	Age 5–9	121.9 (28.5)	138.9 (24.2)	107.8 (25.3)	130.8 (23.8)
	Age 10–14	251.3 (54.9)	235.5 (39.6)	257.7 (63.5)	266.4 (49.3)
	Age 15–<19	334.4 (65.0)	337.3 (45.4)	346.5 (77.0)	285.9 (32.2)
ADHD (*n* = 612,598)					
All		8168.0 (894.0)	8413.2 (876.4)	8116.0 (899.8)	7767.7 (809.8)
Sex	Male	5984.5 (716.7)	6367.7 (662.9)	5873.1 (655.8)	5463.6 (620.0)
	Female	2183.5 (268.1)	2045.6 (228.0)	2243.0 (276.4)	2304.1 (200.9)
Age	Age 0–4	490.0 (131.1)	490.7 (135.8)	502.5 (134.1)	445.2 (108.4)
	Age 5–9	3968.8 (570.5)	4122.5 (590.7)	3939.3 (541.0)	3707.5 (559.5)
	Age 10–14	2604.5 (274.4)	2794.0 (251.2)	2540.2 (227.6)	2378.5 (205.8)
	Age 15–<19	1104.7 (174.2)	1006.1 (107.9)	1134.0 (188.1)	1236.6 (130.3)
ASD (*n* = 872,260)					
All		11,630.1 (1061.0)	11,733.4 (894.8)	11,687.3 (1173.2)	11,188.6 (990.4)
Sex	Male	8008.2 (773.2)	8398.8 (649.9)	7919.1 (753.9)	7393.0 (649.6)
	Female	3621.9 (437.3)	3334.6 (260.4)	3768.2 (462.7)	3795.5 (352.7)
Age	Age 0–4	2348.8 (440.0)	2436.7 (437.2)	2379.5 (426.9)	2034.8 (385.3)
	Age 5–9	4554.4 (386.0)	4636.1 (344.5)	4523.4 (401.8)	4468.6 (423.8)
	Age 10–14	3237.3 (304.8)	3255.2 (266.9)	3258.8 (343.1)	3120.5 (236.9)
	Age 15–<19	1489.7 (230.6)	1405.4 (192.2)	1525.6 (258.2)	1564.6 (157.4)

Values are expressed as mean (standard deviation).

ADHD, attention‐deficit/hyperactivity disorder; ASD, autism spectrum disorder.

For anxiety/stress‐related disorders, no significant overall level changes were observed, but upward trends were shown during restrictions. Initial responses to the pandemic‐related school closures varied across demographic groups. Children aged 5–9 years showed a 12.0% immediate decrease in newly recorded diagnosis cases (Level‐change 1: IRR 0.880, 95% CI: 0.803–0.965). For the overall population during the restriction period, a slight upward trajectory was observed, though it did not reach statistical significance after FDR correction (Slope‐change 1: IRR 1.007, 95% CI: 1.000–1.014, *q = 0.133*). Visual inspection suggested apparent surge patterns coinciding with major COVID‐19 infection waves, particularly during the first 2 years. No significant trend changes were observed in the post‐restriction period (Table 2, Fig. 1, Table S2).

**Table 2:** Baseline incidence rates of newly recorded diagnoses and estimated changes in diagnostic trends across pandemic phases: Interrupted time series analysis.

Mental disorder	Baseline incidence rate	Baseline trend	Level‐change 1 (March 2020; School closure)	Slope‐change 1 (Restriction period)	Level‐change 2 (May 2023; Category V reclassification)	Slope‐change 2 (post‐restriction)
Anxiety/Stress‐related disorders	1.032 (0.979, 1.088)	0.999 (0.996, 1.002)	0.953 (0.829, 1.096)	1.007 (1.000, 1.014)	0.906 (0.805, 1.020)	0.988 (0.975, 1.002)
Mood disorders	0.279 (0.260, 0.298)	1.004 (1.001, 1.008)	1.053 (0.868, 1.278)	1.005 (0.996, 1.015)	0.861 (0.737, 1.006)	0.982 (0.964, 1.000)
Eating disorders	0.043 (0.041, 0.046)	0.996 (0.992, 1.000)	1.090 (0.910, 1.307)	1.004 (0.995, 1.012)	1.064 (0.912, 1.241)	0.986 (0.969, 1.003)
ADHD	0.353 (0.336, 0.371)	1.000 (0.998, 1.003)	0.919 (0.847, 0.997)	1.004 (0.999, 1.008)	0.994 (0.938, 1.053)	0.982 (0.974, 0.990)^†^
ASD	0.505 (0.489, 0.522)	1.000 (0.998, 1.002)	0.959 (0.899, 1.024)	1.004 (1.000, 1.007)	1.014 (0.961, 1.069)	0.981 (0.973, 0.988)^†^

Values are expressed as estimates (95% confidence intervals). Baseline incidence rate represents the observed rate of newly recorded diagnoses (per 1000 person‐months) at the start of the observation period. Baseline trend represents the monthly trend in the pre‐restriction period. Level‐/Slope‐change represents estimated immediate shifts and changes in trends, respectively, at each transition point.

^†^ *q* < 0.05 after false discovery rate correction (Benjamini–Hochberg method).

Abbreviations: ADHD, attention‐deficit/hyperactivity disorder; ASD, autism spectrum disorder.

Population offsets for the overall and 15–<19 years baseline rates are based on 5‐year age‐band estimates and thus include 19‐year‐olds, which is expected to modestly underestimate these baseline rates; level‐ and slope‐change estimates are unaffected.

**Fig. 1:**
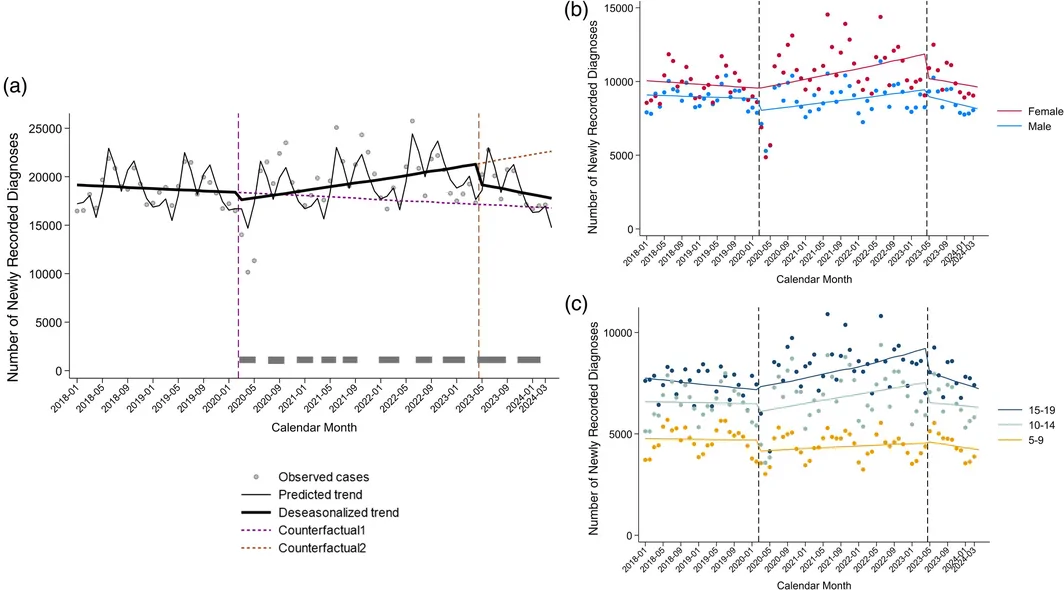
Monthly newly recorded diagnosis cases of anxiety/stress‐related disorders among Japanese children and adolescents (January 2018–March 2024). (a) Overall Trends and Counterfactual Projections. Gray dots, observed monthly counts; thin black lines, predicted monthly rate of newly recorded diagnosis cases; thick black lines, deseasonalized trend. The purple dashed line (Counterfactual 1) represents the projected trend assuming no pandemic onset in March 2020. The brown dashed line (Counterfactual 2) represents the projected trend assuming the pandemic restriction phase had continued beyond May 2023. Dark gray bars along the horizontal axis indicate periods of major COVID‐19 infection waves. (b) Sex‐stratified Trends. Monthly newly recorded diagnosis cases for females (red) and males (blue). (c) Age‐stratified Trends. Trends are categorized by age groups: 5–9 years (orange), 10–14 years (light green), and 15–<19 years (dark blue). Vertical lines: The first vertical dashed line indicates the nationwide school closures (March 2020), and the second indicates the reclassification of COVID‐19 to Category V (May 2023). All models are adjusted for monthly seasonality.

Mood disorders maintained pre‐pandemic upward trends, with emerging signs of recovery post‐restriction in younger groups. Among young children (aged 5–9 years), a 12.2% immediate decrease in newly recorded diagnosis cases was observed at the initial school closures (Level‐change 1: IRR 0.878, 95% CI: 0.770–1.001), although it did not reach statistical significance. In the sex‐stratified analyses, females showed a 17.0% immediate decrease at the lifting of restrictions (Level‐change 2: IRR 0.830, 95% CI: 0.693–0.994), although this did not reach statistical significance after FDR correction (*q = 0.120*). In the age‐stratified analyses, during the post‐restriction period, downward trends were observed in children aged 5–9 years (Slope‐change 2: IRR 0.985, 95% CI: 0.974–0.996) and 10–14 years (Slope‐change 2: IRR 0.976, 95% CI: 0.961–0.992) (Table 2, Fig. 2, Table S2).

**Fig. 2:**
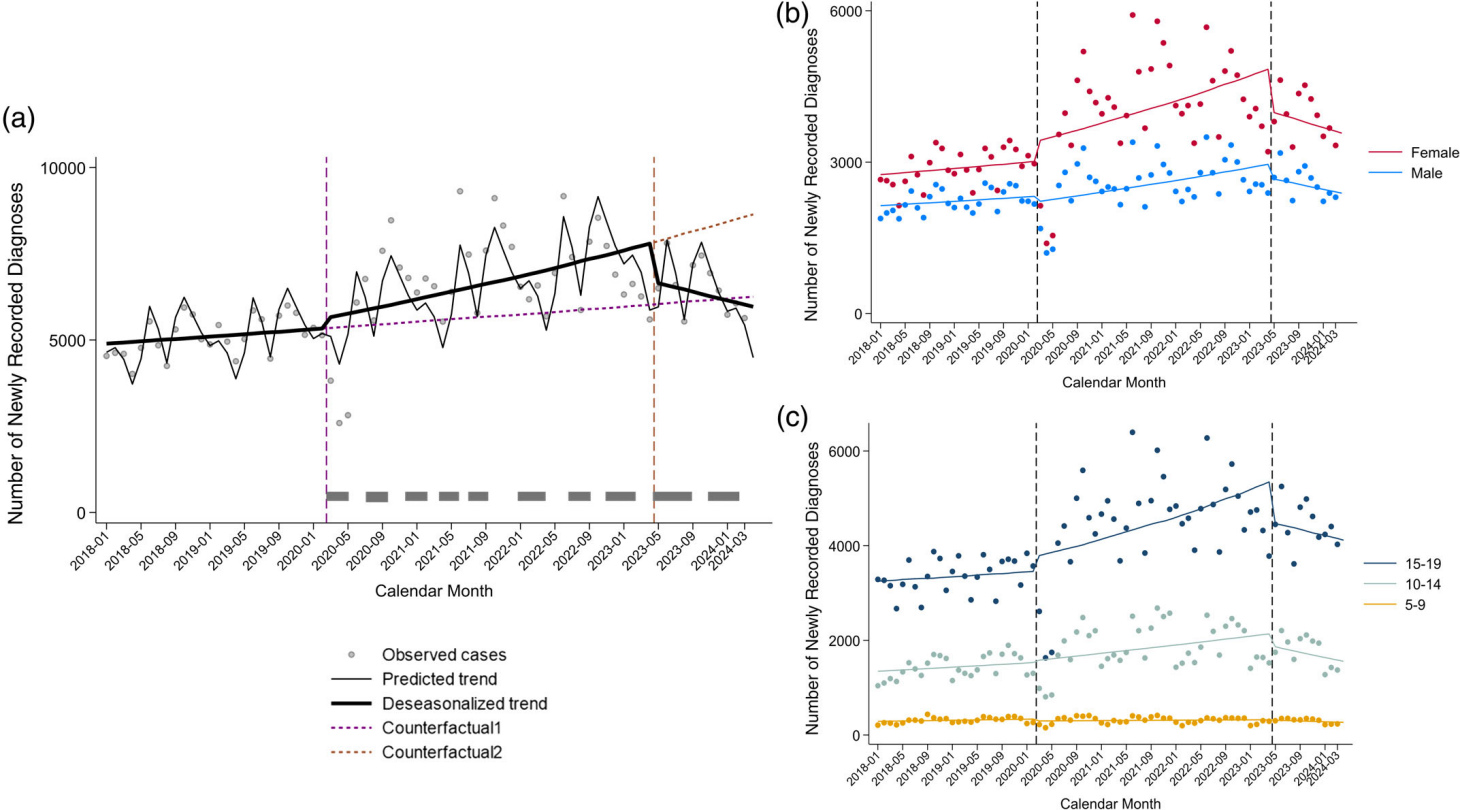
Monthly newly recorded diagnosis cases of mood disorders among Japanese children and adolescents (January 2018 – March 2024). (a) Overall Trends and Counterfactual Projections. Gray dots, observed monthly counts; thin black lines, predicted monthly rate of newly recorded diagnosis cases; thick black lines, deseasonalized trend. The purple dashed line (Counterfactual 1) represents the projected trend assuming no pandemic onset in March 2020. The brown dashed line (Counterfactual 2) represents the projected trend assuming the pandemic restriction phase had continued beyond May 2023. Dark gray bars along the horizontal axis indicate periods of major COVID‐19 infection waves. (b) Sex‐stratified Trends. Monthly newly recorded diagnosis cases for females (red) and males (blue). (c) Age‐stratified Trends. Trends are categorized by age groups: 5–9 years (orange), 10–14 years (light green), and 15–<19 years (dark blue). Vertical lines: The first vertical dashed line indicates the nationwide school closures (March 2020), and the second indicates the reclassification of COVID‐19 to Category V (May 2023). All models are adjusted for monthly seasonality.

Eating disorders exhibited the largest sex‐ and age‐specific divergence among all diagnostic categories. Although the overall population analysis did not reach statistical significance, subgroup patterns were pronounced. At the initial school closures, females showed a 28.4% increase (Level‐change 1: IRR 1.284, 95% CI: 1.045–1.576), with adolescents aged 15–<19 years also showing a 31.6% increase (IRR 1.316, 95% CI: 1.051–1.648); however, neither reached statistical significance after FDR correction (*q* = 0.073 for both). In contrast, males and children aged 5–9 years showed 28.8% and 29.1% decreases, respectively (IRR 0.712, 95% CI: 0.619–0.819 and IRR 0.709, 95% CI: 0.612–0.822). During the restriction period, both males and the 5–9 years age group showed progressive increases (Slope‐change 1: IRR 1.012, 95% CI: 1.004–1.020 for males; IRR 1.016, 95% CI: 1.007–1.024 for ages 5–9 years). In the post‐restriction period, males demonstrated an immediate 21.4% increase (Level‐change 2: IRR 1.214, 95% CI: 1.076–1.370), followed by a declining trend (Slope‐change 2: IRR 0.981, 95% CI: 0.965–0.997), whereas the trend in females remained stable (Table 2, Fig. 3, Table S2).

**Fig. 3:**
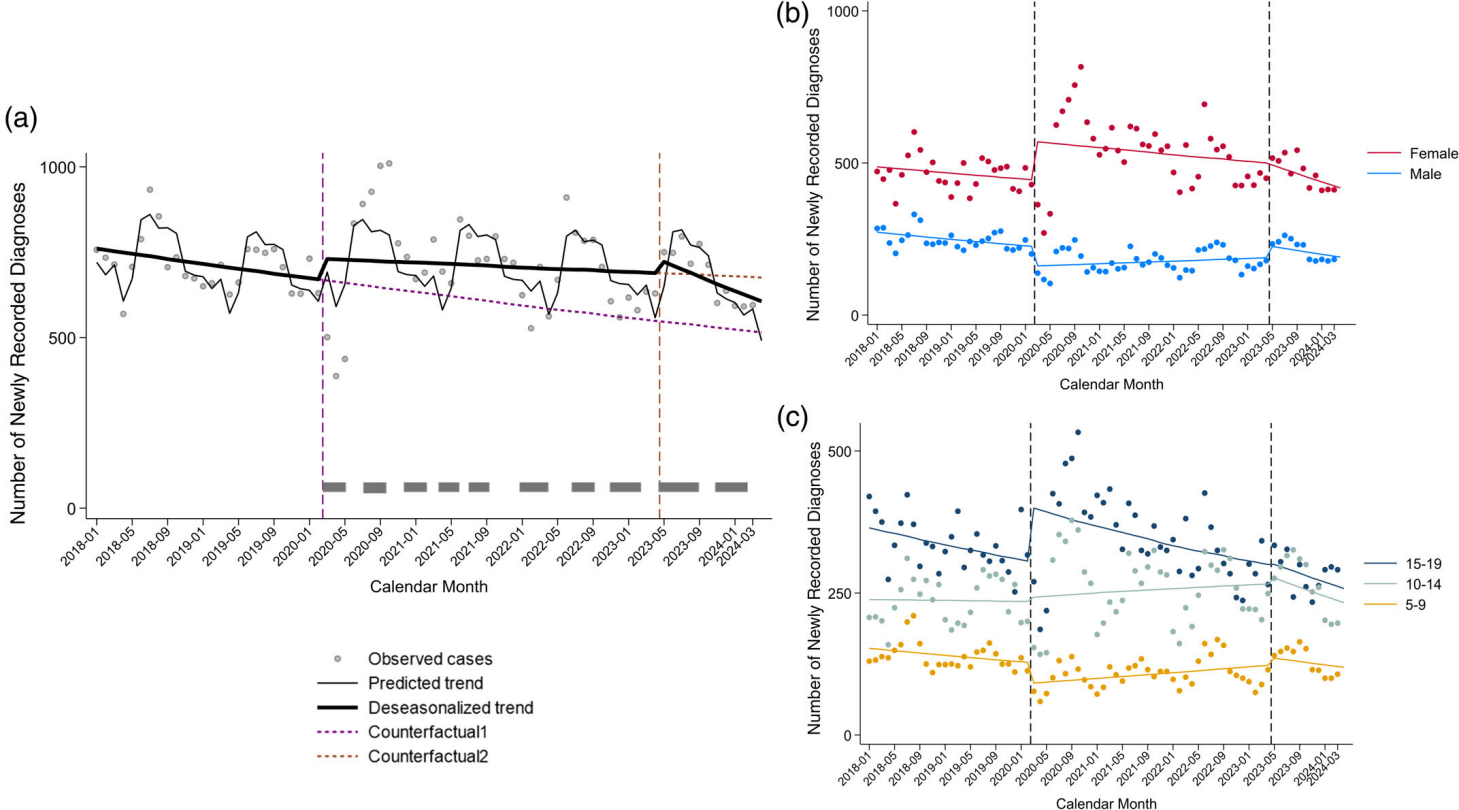
Monthly newly recorded diagnosis cases of eating disorders among Japanese children and adolescents (January 2018 – March 2024). (a) Overall Trends and Counterfactual Projections. Gray dots, observed monthly counts; thin black lines, predicted monthly rate of newly recorded diagnosis cases; thick black lines, deseasonalized trend. The purple dashed line (Counterfactual 1) represents the projected trend assuming no pandemic onset in March 2020. The brown dashed line (Counterfactual 2) represents the projected trend assuming the pandemic restriction phase had continued beyond May 2023. Dark gray bars along the horizontal axis indicate periods of major COVID‐19 infection waves. (b) Sex‐stratified Trends. Monthly newly recorded diagnosis cases for females (red) and males (blue). (c) Age‐stratified Trends. Trends are categorized by age groups: 5–9 years (orange), 10–14 years (light green), and 15–<19 years (dark blue). Vertical lines: The first vertical dashed line indicates the nationwide school closures (March 2020), and the second indicates the reclassification of COVID‐19 to Category V (May 2023). All models are adjusted for monthly seasonality.

Neurodevelopmental disorders showed divergent initial responses—immediate ADHD decline *versus* gradual ASD increase—but converged toward downward trends after restrictions were lifted. Newly recorded ADHD cases showed an immediate 8.1% decrease at the initial school closures (Level‐change 1: IRR 0.919, 95% CI: 0.847–0.997, *q* = 0.120). Although this overall shift did not maintain formal statistical significance under the conservative FDR threshold, the downward trend was observed and was highly significant within specific demographic strata, most notably among children aged 10–14 years (IRR 0.879, 95% CI: 0.819–0.944, *q* < 0.001). During the restriction period, upward trends were observed among adolescents aged 15–<19 years (Slope‐change 1: IRR 1.012, 95% CI: 1.006–1.018, *q* < 0.001). In the post‐restriction period, downward trends reaching statistical significance were observed across nearly all subgroups (Slope‐change 2: IRR 0.982, 95% CI: 0.974–0.990) (Table 2, Fig. 4, Table S2).

**Fig. 4:**
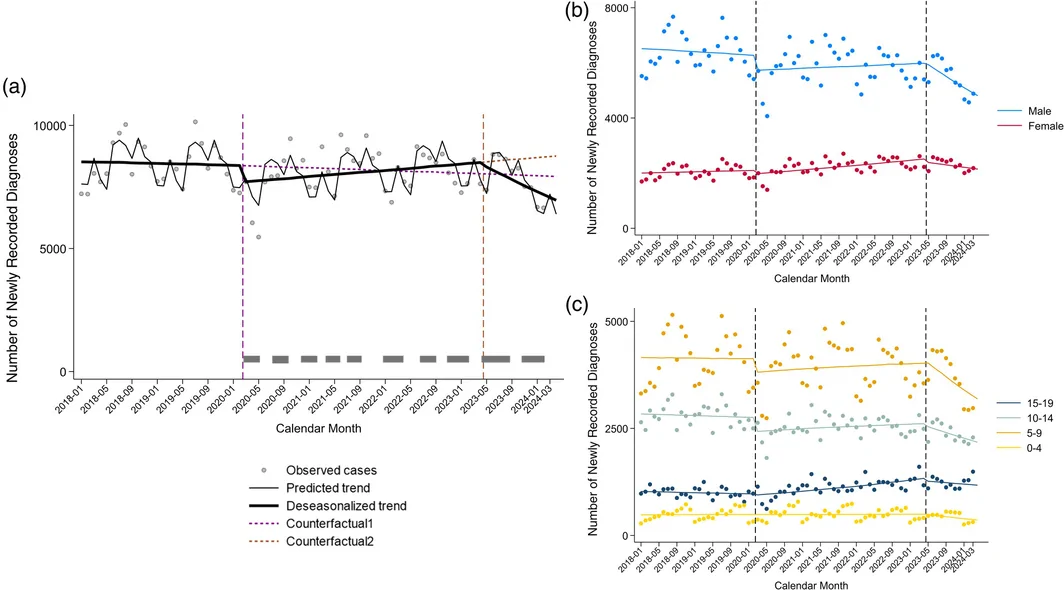
Monthly newly recorded diagnosis cases of ADHD among Japanese Children and Adolescents (January 2018 – March 2024). (a) Overall Trends and Counterfactual Projections. Gray dots, observed monthly counts; thin black lines, predicted monthly rate of newly recorded diagnosis cases; thick black lines, deseasonalized trend. The purple dashed line (Counterfactual 1) represents the projected trend assuming no pandemic onset in March 2020. The brown dashed line (Counterfactual 2) represents the projected trend assuming the pandemic restriction phase had continued beyond May 2023. Dark gray bars along the horizontal axis indicate periods of major COVID‐19 infection waves. (b) Sex‐stratified Trends. Monthly newly recorded diagnosis cases for females (red) and males (blue). (c) Age‐stratified Trends. Trends are categorized by age groups: 0–4 years (yellow), 5–9 years (orange), 10–14 years (light green), and 15–<19 years (dark blue). Vertical lines: The first vertical dashed line indicates the nationwide school closures (March 2020), and the second indicates the reclassification of COVID‐19 to Category V (May 2023). All models are adjusted for monthly seasonality.

Newly recorded ASD cases demonstrated a pattern partly distinct from ADHD, with no significant immediate level change observed at the initial school closures, except for children aged 5–9 years (Level‐change 1: IRR 0.933, 95% CI: 0.885–0.983). ASD cases showed gradual increases during the restriction period (Slope‐change 1: IRR 1.004, 95% CI: 1.000–1.007), with statistically significant increases observed in adolescents aged 15–<19 years (Slope‐change 1: IRR 1.011, 95% CI 1.005–1.016, *q* < 0.001); trends in the same direction were observed among females and younger children, though these did not reach statistical significance after FDR correction. Consistent with ADHD, downward trends reaching statistical significance were observed across all demographic subgroups in the post‐restriction period (Slope‐change 2: IRR 0.981, 95% CI: 0.973–0.988) (Table 2, Fig. 5, Table S2).

**Fig. 5:**
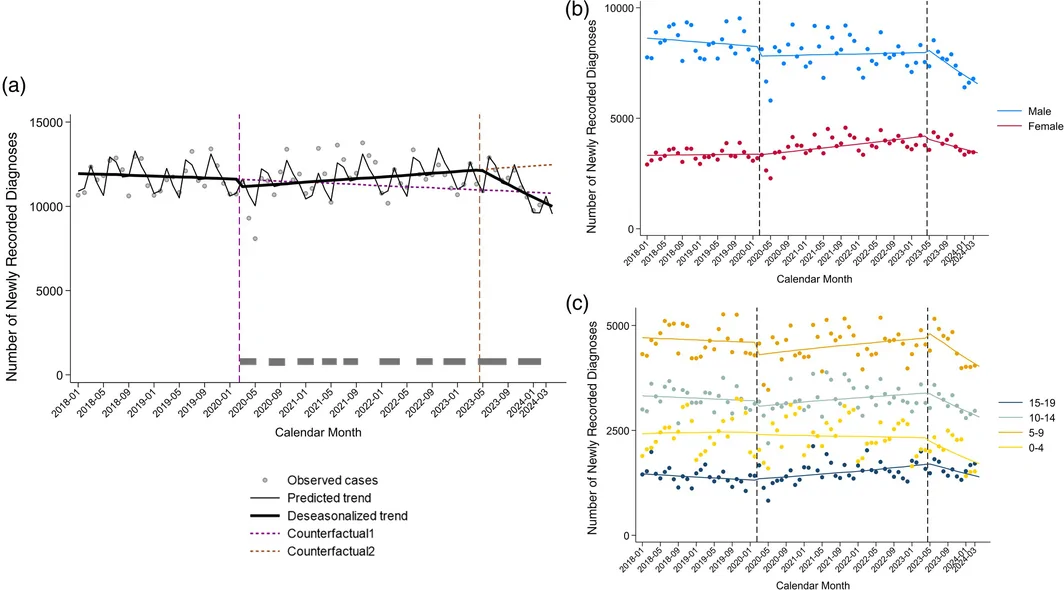
Monthly newly recorded diagnosis cases of ASD among Japanese children and adolescents (January 2018 – March 2024). (a) Overall Trends and Counterfactual Projections. Gray dots, observed monthly counts; thin black lines, predicted monthly rate of newly recorded diagnosis cases; thick black lines, deseasonalized trend. The purple dashed line (Counterfactual 1) represents the projected trend assuming no pandemic onset in March 2020. The brown dashed line (Counterfactual 2) represents the projected trend assuming the pandemic restriction phase had continued beyond May 2023. Dark gray bars along the horizontal axis indicate periods of major COVID‐19 infection waves. (b) Sex‐stratified Trends. Monthly newly recorded diagnosis cases for females (red) and males (blue). (c) Age‐stratified Trends. Trends are categorized by age groups: 0–4 years (yellow), 5–9 years (orange), 10–14 years (light green), and 15–<19 years (dark blue). Vertical lines: The first vertical dashed line indicates the nationwide school closures (March 2020), and the second indicates the reclassification of COVID‐19 to Category V (May 2023). All models are adjusted for monthly seasonality.

## Discussion

Using national NDB data, we examined long‐term trajectories of newly recorded pediatric psychiatric disorders across two pandemic phases. Our findings suggest that patterns of new diagnoses may reflect two complementary mechanisms: (i) acute stress responses, with mood and anxiety/stress‐related disorders showing immediate reactions to pandemic‐related distress, and (ii) shifts in person–environment fit, with neurodevelopmental disorders reflecting changing dynamics between individual characteristics and environmental demands. Eating disorders exhibited patterns that partially overlap with both mechanisms. By focusing on newly recorded cases, these results provide insights into shifts in the clinical thresholds for mental health‐related contact during the pandemic. Diagnostic trajectories diverged into three broad patterns, discussed below by category.

### Healthcare access and clinical contact

An important consideration in interpreting these trajectories is the potential influence of pandemic‐related changes in healthcare‐seeking behavior. A nationwide NDB study reported an approximately 10% reduction in overall outpatient visits in 2020,^23^ and national data confirmed that volumes normalized by late 2021.^24^ Telepsychiatry, unlike in Western countries, remains limited in Japan owing to systemic and reimbursement barriers.^25^ Initial decreases in newly recorded diagnoses may therefore partly reflect reduced healthcare contact. Importantly, this supply‐side reduction — driven by infection avoidance rather than clinical need — would have affected all diagnostic categories similarly, and thus cannot explain the divergent trajectories observed. The divergence is more plausibly attributable to demand‐side, diagnosis‐specific mechanisms, including differential patterns of symptom emergence (e.g., acute stress responses or person–environment fit shifts) and changes in detection or referral pathways (e.g., reduced school‐based identification of ADHD during closures), as discussed below.

### Mood disorders, anxiety/stress‐related disorders and social connectivity

The dynamics of mood disorders and anxiety/stress‐related disorders in Japan showed both commonalities with and deviations from international trends. A global meta‐analysis reported a doubling in the prevalence of clinically elevated depressive and anxiety symptoms during the pandemic^1^. In contrast, newly recorded cases in Japanese children did not show notable increases overall. Although our mood disorder category includes bipolar‐spectrum codes (F30–F31), these accounted for only 12.5% of cases, whereas 76.8% were recorded as single depressive episodes (F329: depressive episode, unspecified) (Table S1); thus, the observed trajectories predominantly reflect depressive dynamics. Both mood and anxiety/stress‐related disorders exhibited substantial month‐to‐month variability during the restriction period, with apparent surges following each COVID‐19 infection wave, particularly during the first 2 years. The initial surge following the first and second waves (spring and summer 2020) appeared to reflect delayed healthcare‐seeking after temporary medical visit avoidance, and subsequent surges continued to occur following later waves. This variability may reflect both the psychological effects of infection waves and delayed healthcare‐seeking during infection peaks. For mood disorders, the pre‐existing upward trend, combined with large variability and reliance on clinical diagnoses rather than symptom scales, may have obscured pandemic‐related increases. The observed 17.0% immediate decrease in female diagnoses after restrictions lifted, and the downward trends among children aged 5–14 years, suggest a recovery process potentially driven by restored social connections, consistent with the US Youth Risk Behavior Survey Data findings^26^ and Malaysian reports indicating that elementary students viewed school resumption more positively,^27^ and support the hypothesis that restoration of school functions acted as a protective factor for younger age groups. In contrast, the 15–<19 years age group did not show an equivalent post‐restriction recovery. Unlike children in compulsory education, this age group encompasses diverse educational and occupational settings with heterogeneous stressor profiles and access to mental health services, which may explain the absence of a clear recovery pattern.

### Sex‐ and age‐specific patterns in eating disorders

Eating disorder trajectories showed sex‐ and age‐specific patterns. The 28.4% surge in case detection in females following school closures aligns with systematic reviews suggesting social isolation and routine disruption as contributing factors.^28^, ^29^ In contrast, males and children aged 5–9 years showed an immediate 28.8% decrease and a 29.1% decrease following school closures, respectively, with subsequent gradual increases during restrictions and an immediate 21.4% increase in males in the post‐restriction period. Unlike mood disorders and anxiety/stress‐related disorders, which showed repeated surge patterns, eating disorders exhibited a similar surge concentrated in the initial pandemic phase, which may reflect medical visit avoidance and subsequent rebound. The convergent patterns of males and young children warrant attention. This demographic profile (younger age and relatively higher male presentation) is consistent with the epidemiological features of avoidant/restrictive food intake disorder (ARFID),^30^ although our administrative data cannot differentiate ARFID from other eating disorder subtypes, and coding‐based limitations for this category warrant caution (see Limitations). The resumption of communal meals after prolonged *moku‐shoku* (“silent lunches”) practices may have contributed to both increased detection and environmental triggers in susceptible individuals, though this interpretation requires validation with clinical cohort data.^31^

### Neurodevelopmental disorders and person–environment interactions

Neurodevelopmental disorders showed trajectories suggesting person–environment interactions. The initial 8.1% decrease in new ADHD diagnoses, followed by the absence of a compensatory rebound, contrasts with the gradual increase observed for ASD during the same restriction period. Because healthcare avoidance would have affected both conditions similarly, this divergence suggests that differences in how environmental demands interact with each condition's symptom profile^15^—rather than differential healthcare access—played a more prominent role. Specifically, the removal of structured school routines may have reduced the visibility of hyperactive and inattentive symptoms that typically prompt ADHD referral,^32^, ^33^ whereas disrupted routines and mask‐related communication barriers may have progressively exacerbated difficulties for children with ASD.^34^ Following the May 2023 reclassification, both conditions showed preliminary downward trends. Although the observation period is limited, the absence of post‐restriction rebound during this period is consistent with the hypothesis that pandemic‐period fluctuations were mainly driven by environmental pressures rather than delayed consultations. These post‐restriction downward trends may reflect multiple non‐mutually exclusive mechanisms. Japan's GIGA School Program, which accelerated the deployment of one digital device per student in all public elementary and junior high schools by March 2021,^35^ may have diversified learning environments in ways that reduced person–environment mismatches for some children, though the specific impact on neurodevelopmental disorder identification remains unknown. Alternatively, the gradual increase during the restriction period may have partly depleted the pool of undiagnosed susceptible individuals, contributing to the subsequent decline. The relative contribution of these mechanisms cannot be determined from the present data.

These contrasting subgroup‐level trajectories have a practical implication. Analysis of the total population alone would have masked opposing shifts occurring simultaneously, such as emerging increases in eating disorder diagnoses among males alongside declining neurodevelopmental disorder diagnoses during the same post‐restriction period. Such divergence highlights the potential value of routine psychiatric surveillance systems capable of tracking youth diagnostic trends by disorder, sex, and age. However, the present study cannot directly inform decisions about clinical resource reallocation, as it does not capture severity, care utilization, or unmet need. The primary value of these findings is to identify where further targeted research is warranted — including prospective clinical registries or claims‐linked studies that can distinguish between increased underlying incidence, deferred consultations, and rebound presentations.

### Strengths

The strengths of this study are threefold. First, the NDB covers over 99% of healthcare claims in Japan,^14^ minimizing the sampling bias inherent in smaller cohorts and providing sufficient statistical power to examine demographic subgroups across Japan. Furthermore, this near‐census coverage means that the NDB approximates the target population rather than a sample drawn from it; consequently, observed differences directly reflect population‐level changes rather than sampling variability. In this context, statistical tests and confidence intervals reflect model‐based uncertainty from the segmented regression rather than classical sampling error. Although FDR correction was applied conservatively to address multiple comparisons across diagnostic categories and subgroups, both FDR‐adjusted *q*‐values and unadjusted *P*‐values are reported in Table S2 to allow readers to apply their preferred inferential threshold. Second, the simultaneous analysis of five diagnostic categories within a single national dataset enables direct within‐study comparison of divergent trajectories driven by shared environmental exposures — a cross‐diagnostic design that allows contrasting mechanisms (acute stress response *versus* person–environment fit) to be examined within the same population, sex, and age strata. Third, the ITS design leverages two clearly defined policy transitions — the March 2020 school closures and the May 2023 Category V reclassification — as natural experiments, enabling estimation of both immediate level shifts and longer‐term trend changes without requiring individual‐level randomization. The May 2023 reclassification represented a legally defined, simultaneous termination of school‐based restrictions, providing a distinct transition point for evaluating post‐restriction trajectories.

### Limitations

Nevertheless, several limitations warrant consideration.

First, a patient's diagnosis start date may reset after a change in health insurance provider or a transfer between institutions, potentially causing re‐registrations to be misclassified as new cases. Because our claims data extraction covered the period from 2017 onward, this risk was especially high immediately afterward. We addressed it *via* a 12‐month washout period (2017). Although this risk likely dilutes over time, the same event can still occur after the washout period and may have been heightened during the pandemic, potentially leading to an overcount of diagnoses.

Second, the outcomes reflect first recorded psychiatric diagnoses in administrative claims data, not true disease incidence. The timing of a first recorded diagnosis may lag behind symptom onset due to delays in help‐seeking and evaluation, and pandemic‐related changes in healthcare‐seeking behavior, awareness, and diagnostic thresholds may influence recorded rates independently of true disorder occurrence. Our case definition also cannot distinguish a newly identified diagnosis during ongoing non‐psychiatric care, an additional diagnosis during ongoing psychiatric treatment, or a first specialty visit. Furthermore, the processes underlying symptom emergence, help‐seeking, clinical decision‐making, and reimbursement coding cannot be disentangled in the present analysis. Time‐stable sources of measurement variation would affect the baseline uniformly and are unlikely to account for the bidirectional shifts observed at two distinct policy transitions. However, these mechanisms cannot be entirely excluded, and trajectories should be interpreted as reflecting changes in clinically recognized and recorded disorders rather than in underlying disease occurrence.

Third, administrative claims data lack severity scales and detailed psychosocial context. Although “suspected” diagnoses were excluded, reimbursement‐driven coding may not strictly align with clinical criteria,^36^ and specific subtypes such as ARFID cannot be accurately identified. Similarly, non‐specific codes such as F50.9 (67.8% of eating disorder cases; Table S1) may occasionally reflect general feeding or swallowing concerns rather than a confirmed diagnosis (Methods S2). Sensitivity analyses restricted to primary diagnoses were not conducted owing to inconsistent coding practices across NDB settings. The ITS design relies on the consistency of coding practices over time rather than its absolute accuracy, so stable coding preferences would affect the series level uniformly and are unlikely to explain the direction‐changing shifts at two distinct transitions. Mood disorder codes may also reflect off‐label prescribing (e.g., sleep disturbance) rather than a primary psychiatric diagnosis,^36^ a bias that exclusion of ‘suspected’ diagnoses cannot fully correct. Findings for mood disorders should therefore be interpreted with particular caution. Procedural‐level reimbursement data, including psychotropic prescriptions and psychiatric specialty therapy fees, were unavailable in the current data application and should be incorporated in future research.^33^

Fourth, the sequential nature of psychiatric diagnosis warrants consideration. Children with neurodevelopmental disorders frequently present initially with anxiety or mood symptoms as their chief complaint, and the neurodevelopmental diagnosis may only be recorded at a subsequent visit after further evaluation. A proportion of “new” neurodevelopmental disorder diagnoses in our data may therefore reflect a delayed second‐step recording rather than a first clinical contact, and the timing of this delay may have varied across pandemic phases depending on the availability and continuity of specialty evaluation. This mechanism may have attenuated or distorted the apparent timing of changes in neurodevelopmental disorder diagnoses and should be considered when interpreting the divergent trajectories between mood and anxiety/stress‐related disorders and neurodevelopmental disorders. Similarly, our independent analysis of each diagnostic category does not account for potential interactions among comorbid conditions, as initial recognition of one disorder may prompt evaluation and subsequent diagnosis of comorbid conditions, creating temporal dependencies that our models do not capture.

Fifth, as noted above, pandemic‐related medical visit avoidance may have caused temporary under‐identification. However, because this mechanism would affect all categories uniformly, it cannot explain the divergent, category‐specific trajectories central to this study.

Sixth, the 11‐month post‐restriction period may be insufficient to differentiate sustained slope changes from transient post‐transition adjustments, and early trend estimates should be interpreted as preliminary. This window does not allow us to determine whether the downward trends in neurodevelopmental disorder diagnoses reflect a lasting reduction in person–environment mismatch following school resumption, or a transient stabilization after restriction‐period accumulation, nor whether the absent rebound in female eating disorder diagnoses represents a true plateau or a delayed increase. Distinguishing these possibilities will require extended observation from fiscal year 2024 onward, ideally alongside clinical cohort data providing individual‐level symptom trajectories independent of healthcare‐seeking behavior. Subgroup analyses more broadly should be interpreted as hypothesis‐generating, emphasizing the magnitude and cross‐subgroup consistency of effect estimates over dichotomous significance thresholds.

Finally, this study cannot distinguish the effects of pandemic‐related social policies from the potential neuropsychiatric sequelae of SARS‐CoV‐2 infection itself, which have been reported in pediatric populations, nor can ITS analysis definitively attribute observed shifts to specific pandemic policies owing to the potential influence of unmeasured confounding factors, including broader socioeconomic changes during the study period.

## Conclusions

The COVID‐19 pandemic and subsequent lifting of restrictions were associated with heterogeneous changes in newly recorded pediatric psychiatric disorders in Japan. Mood disorders and anxiety/stress‐related disorders showed repeated surge patterns following infection waves, particularly during the initial pandemic years, without clear sustained increases. Eating disorders exhibited sex‐ and age‐specific patterns, with immediate female increases following school closures *versus* later increases among males and young children following the lifting of restrictions, with variability concentrated in the initial pandemic phase. Neurodevelopmental disorders showed divergent initial responses with preliminary downward trends in the post‐restriction period, suggesting a central role of environmental demands. These contrasting trajectories across disorders, sex, and age highlight the importance of routine psychiatric surveillance with subgroup‐level granularity. Whether these changes represent transient reactions or sustained shifts remains to be determined with longer post‐restriction follow‐up.

## Author contributions

N.M. Conceptualization, study design, data acquisition/verification, statistical analysis, interpretation, literature search, and drafting. S.H. Data acquisition, cleaning, and statistical analysis. Y.S., A.O., T.A., and T.H. Interpretation, literature search, and critical revision. H.I. Critical revision. T.Y. Supervision and critical revision. All authors approved the final manuscript. N.M. and S.H. verified the underlying data. N.M. has final responsibility for submission.

## Disclosure statement

The authors have no conflicts of interest to declare.

## Supporting information


**Methods S1.** Additional details on data source and study population.
**Methods S2.** Diagnostic validity and categorization.
**Methods S3.** Statistical model specification and interpretation of estimates.

**Table S1.** Claims‐level disease codes used to identify cases.
**Table S2.** Baseline incidence rates and estimated changes in new diagnostic trends for mental disorders across pandemic phases: Overall and subgroup‐specific interrupted time‐series analysis.

## Data Availability

The National Database of Health Insurance Claims (NDB) data are maintained by the Ministry of Health, Labour and Welfare, Japan, and cannot be shared due to legal restrictions on patient privacy. Data on COVID‐19 infection trends were obtained from publicly available sources: the Japan Institute for Health Security (JIHS; https://id-info.jihs.go.jp/) and the Ministry of Health, Labour and Welfare (https://www.mhlw.go.jp/index.html). The analysis code is available from the corresponding author upon reasonable request.
