# Supplementary material for: Newly recorded psychiatric diagnoses among 21 million youth before, during, and after COVID‐19 restrictions: A nationwide interrupted time‐series analysis
Source: Psychiatry Clin Neurosci. 2026 Aug 10;80(11):905–14. doi: 10.1111/pcn.70117 (PMC13732791; doi:10.1111/pcn.70117)
Supplement: Supplementary file 1 [file PCN-80-905-s001.docx]

**Supplementary Methods**

*Newly Recorded Psychiatric Diagnoses Among 21 Million Youth Before, During, and After COVID-19 Restrictions: A Nationwide Interrupted Time-Series Analysis*

**S1. Additional Details on Data Source and Study Population**

Patient records in the NDB are linked longitudinally using a pseudonymized identifier derived from the insured person’s identification number through a one-way hashing process, in accordance with the anonymization standards established by the Ministry of Health, Labour and Welfare. For each individual, the date of first diagnosis was determined from the diagnosis start date recorded for the target ICD-10 code in the claims data. According to the Statistics Bureau, the age-stratified population as of March 2020 in Japan was as follows: 4.65 million (0–4 years), 5.14 million (5–9 years), 5.37 million (10–14 years), and 5.81 million (15–19 years).^1^ We targeted children and adolescents aged under 19 years. The study period was initiated in January 2018 to provide a pre-pandemic baseline of at least 26 months, following a 12-month washout period in 2017, which offers more than two full annual cycles necessary for stable trend estimation and seasonal adjustment within the Japanese academic calendar.

**S2. Diagnostic Validity and Categorization**

We included only confirmed diagnoses and excluded all records flagged as “suspected” to maintain high diagnostic specificity. Based on clinical stability and typical onset age, we applied the following age-based exclusion criteria and specific International Statistical Classification of Diseases and Related Health Problems, Tenth Revision (ICD-10), codes and Japanese claims codes to align with modern clinical standards (Diagnostic and Statistical Manual of Mental Disorders, Fifth Edition, diagnoses).

For mood disorders (ICD-10 F30–F39), anxiety/stress-related disorders (F40–F48), and eating disorders (F50), we restricted inclusion to ages 5–<19 years (excluding 0–4 years). This F50-based definition is broader than, and should not be equated with, specialist-diagnosed anorexia nervosa or bulimia nervosa based on structured clinical criteria. ICD-10 provides more specific codes for feeding problems in infancy and childhood (e.g., feeding problems of infancy and childhood, F98.2; unspecified feeding difficulties, R63.3), which are most likely to be confused with F50 codes; although our study population already excludes infancy (0–4 years), some degree of this misclassification cannot be ruled out among the ages analyzed. Non-specific codes such as F50.9 (67.8% of eating disorder cases; Supplementary Table S1) may also occasionally reflect general feeding or swallowing concerns rather than a confirmed diagnosis.

For neurodevelopmental disorders (ADHD and ASD), we included the full pediatric age range (under 19 years). ADHD was defined by F90.0, F90.1, F90.9, and F98.8 codes. We included the Japanese claims code “attention deficit disorder without hyperactivity” (Code: 8836994; mapped to F98.8) to capture predominantly inattentive presentations, while excluding non-specific behavioral codes (Code: 8834784). ASD was identified using the F84 category. We explicitly excluded Rett syndrome (F84.2), *MECP2* duplication syndrome (8849859), and “Childhood type schizophrenia” (8842271) to ensure diagnostic purity. The specific ICD-10 subcategories included within each broad diagnostic group are listed in Supplementary Table 1. Regarding comorbidity, each diagnostic category was analyzed independently. A single patient could be counted in multiple diagnostic categories if they received confirmed diagnoses in more than one category (e.g., a patient with both ADHD and anxiety disorders would be included in both the ADHD and anxiety/stress-related disorder analyses). However, within each diagnostic category, each patient was counted only once, at the time of their first confirmed diagnosis. This approach was chosen because the study’s objective was to examine temporal trends in new clinical recognition for each disorder, rather than to estimate person-level comorbidity patterns.

**S3. Statistical Model Specification and Interpretation of Estimates**

We performed ITS analysis using segmented negative binomial regression.^2^ The model was adjusted for seasonality using month dummy variables, as this approach yielded a lower Akaike information criterion than models using Fourier terms, indicating a superior fit for the discrete seasonal fluctuations in the Japanese academic calendar. This approach allows flexible estimation of month-specific effects without imposing a parametric seasonal structure. Residual autocorrelation was assessed using the Portmanteau test (Box–Ljung Q statistic) applied to Pearson residuals and was found to be significant across all diagnostic models (all p < 0.001); Newey–West HAC standard errors were therefore applied to all models to ensure valid inference in the presence of serial correlation.^3^ To control for time-varying fluctuations in the dynamic population, we obtained monthly population estimates from the Statistics Bureau of Japan and included them as an offset term.^1^ For each analysis, we used the corresponding population (total for overall analyses, sex- and age-specific for stratified analyses) as the offset term. Although some literature suggests that a longer follow-up period is preferable for stable trend estimation, our 11-month post-restriction period exceeds the empirical minimum of eight observations required to statistically estimate immediate level shifts and early trend changes.^2,4^

The primary regression model was specified as follows:

$$\log\left( E\left[ Y_{t} \right] \right)=\beta_{0}+\beta_{1}T_{t}+\beta_{2}D_{1,t}+\beta_{3}P_{1,t}+\beta_{4}D_{2,t}+\beta_{5}P_{2,t}+\sum_{k=2}^{12} \gamma_{k}M_{k,t}+\log\left( \text{Population}_{t} \right)$$

where *Y*_t_ is the monthly newly recorded diagnosis cases; *T*_t_ is the elapsed time in months since the start of the study period (January 2018 = 0); and *D* and *P* represent level and trend shifts for the two interventions, respectively. Specifically, *D*_1,t_ and *D*_2,t_ are indicator variables used to estimate the immediate level shifts in case identification following the initial school closures (March 2020, *D*_1,t_) and the reclassification of COVID-19 as Category V (May 2023, *D*_2,t_), respectively. *D*_2,t_ estimates the additional immediate change relative to the preceding pandemic restriction period. *P*_1,t_ and *P*_2,t_ represent the elapsed time following each intervention to estimate changes in the longitudinal trend (slope-change). Here, *P*_2,t_ captures the shift in the trend compared with the trajectory established during the pandemic restriction phase. To adjust for seasonal fluctuations within the Japanese academic calendar, monthly dummy variables *M*_k,t_ were included.

By exponentiating the coefficients, the results are expressed as incidence rate ratios (IRRs) with 95% confidence intervals (CIs). Of note, the IRRs in this study reflect ratios of newly recorded diagnosis case rates rather than true disease incidence rates. For level-change terms (*D*₁, *D*₂), the IRR represents the immediate multiplicative shift in the monthly rate of new diagnoses at the time of the policy change, relative to the counterfactual rate expected from the preceding trajectory; for example, an IRR of 0.92 for *D*₁ indicates an 8% immediate decrease. For slope-change terms (*P*₁, *P*₂), the IRR represents the multiplicative change in the monthly trend per additional month elapsed after the intervention, relative to the trend in the preceding period.

We first constructed ITS models for the total population, followed by separate analyses for sex and age subgroups. To address potential problems with multiple comparisons across five diagnostic categories and multiple subgroups, the false discovery rate (FDR) was controlled using the Benjamini–Hochberg procedure,^5^ with statistical significance defined as FDR-adjusted *q*-values <0.05; both FDR-adjusted q-values and unadjusted p-values are reported in Supplementary Table 2. All analyses were performed using Stata version 19.0 (StataCorp LLC, College Station, TX, USA).

**References**

1.	Population Estimates Monthly Report Monthly. *Portal Site of Official Statistics of Japan*. [Cited 2026 Jan 3] Available from https://www.e-stat.go.jp/en/stat-search/files?page=1&layout=datalist&toukei=00200524&tstat=000000090001&cycle=1&tclass1=000001011678&tclass2val=0

2.	Bernal JL, Cummins S, Gasparrini A. Interrupted time series regression for the evaluation of public health interventions: a tutorial. *Int. J. Epidemiol.* 2017; 46: 348–55.

3.	Newey WK, West KD. A Simple, Positive Semi-Definite, Heteroskedasticity and Autocorrelation Consistent Covariance Matrix. *Econometrica*. 1987; 55: 703.

4.	Penfold RB, Zhang F. Use of interrupted time series analysis in evaluating health care quality improvements. *Acad. Pediatr.* 2013; 13: S38-44.

5.	Benjamini Y, Hochberg Y. Controlling the false discovery rate: A practical and powerful approach to multiple testing. *J. R. Stat. Soc. Series B Stat. Methodol.* 1995; 57: 289–300.
