# Supplementary material for: Newly recorded psychiatric diagnoses among 21 million youth before, during, and after COVID‐19 restrictions: A nationwide interrupted time‐series analysis
Source: Psychiatry Clin Neurosci. 2026 Aug 10;80(11):905–14. doi: 10.1111/pcn.70117 (PMC13732791; doi:10.1111/pcn.70117)
Supplement: Supplementary file 2 [file PCN-80-905-s002.docx]

| **Supplementary Table S1. Claims-level disease codes used to identify cases.** | | | |  |
| --- | --- | --- | --- | --- |
| **Diagnostic Category** | **Disease Code** | **Disease Name** | **ICD-10** | **Proportion (%)** |
| **Anxiety/stress-related disorders** | 3009007 | Neurosis, unspecified | F489 | 22.79 |
| (F40–F48) | 3000010 | Anxiety neurosis | F411 | 14.69 |
| n = 1,432,284 | 8837899 | Adjustment disorder | F432 | 13.01 |
|  | 8839596 | Anxiety disorder | F419 | 5.56 |
|  | 3069005 | Psychosomatic disorder | F459 | 5.07 |
|  | 8834790 | Childhood psychosomatic disorder | F459 | 4.76 |
|  | 3008007 | Childhood neurosis | F488 | 3.86 |
|  | 3061009 | Hyperventilation syndrome | F453 | 3.76 |
|  | 8846667 | Psychogenic visual impairment | F458 | 2.71 |
|  | 3065006 | Psychogenic pollakiuria | F453 | 2.41 |
|  | 3061004 | Pharyngolaryngeal neurosis | F453 | 2.04 |
|  | 8835104 | Somatoform disorder | F459 | 1.93 |
|  | 7849002 | Pharyngolaryngeal dysesthesia | F453 | 1.53 |
|  | 8832567 | Obsessive-compulsive disorder | F429 | 1.48 |
|  | 8842740 | Social anxiety disorder | F401 | 1.22 |
|  | 3067001 | Psychogenic hearing loss | F446 | 1.09 |
|  | 8845669 | Social anxiety disorder | F401 | 0.98 |
|  | 3000004 | Anxiety-tension state | F411 | 0.90 |
|  | 8834946 | Neurasthenia | F480 | 0.70 |
|  | 8845678 | Post-traumatic stress disorder | F431 | 0.68 |
|  | 8844095 | Panic disorder | F410 | 0.67 |
|  | 3379028 | Autonomic seizure | F445 | 0.46 |
|  | 3078008 | Psychogenic abdominal pain | F454 | 0.43 |
|  | 8836575 | Generalized anxiety disorder | F411 | 0.39 |
|  | 8831076 | Dissociative disorder | F449 | 0.37 |
|  | 3064008 | Aerophagy | F453 | 0.34 |
|  | 3061005 | Pharyngeal dysesthesia | F453 | 0.33 |
|  | 3089002 | Acute stress reaction | F430 | 0.27 |
|  | 3069006 | Psychosomatic autonomic dysfunction | F453 | 0.26 |
|  | 3061017 | Psychogenic cough | F453 | 0.23 |
|  | 3078007 | Psychogenic headache | F454 | 0.23 |
|  | 8830642 | Pharyngolaryngoesophageal neurosis | F453 | 0.22 |
|  | 8833894 | Mixed anxiety and depressive disorder | F412 | 0.22 |
|  | 3067004 | Nervous tinnitus | F458 | 0.21 |
|  | 8835100 | Somatization disorder | F450 | 0.20 |
|  | 3003003 | Obsessional neurosis | F421 | 0.19 |
|  | 7849011 | Nasopharyngeal dysesthesia | F453 | 0.19 |
|  | 8846666 | Psychogenic visual field disorder | F458 | 0.17 |
|  | 8839790 | Vague somatic complaints | F459 | 0.17 |
|  | 3062013 | Cardiac neurosis | F453 | 0.16 |
|  | 3064025 | Neurogenic esophageal stenosis | F453 | 0.15 |
|  | 8846871 | Conversion disorder | F449 | 0.15 |
|  | 3067008 | Functional hearing loss | F446 | 0.15 |
|  | 8844096 | Panic attack | F410 | 0.13 |
|  | 3064022 | Nervous gastroenteritis | F453 | 0.12 |
|  | 3062022 | Nervous palpitation | F453 | 0.12 |
|  | 3009009 | Psychoneurosis | F489 | 0.12 |
|  | 8832574 | Phobic anxiety disorder | F409 | 0.11 |
|  | 3004024 | Anxiety depression | F412 | 0.10 |
|  | 3078012 | Nervous otalgia | F454 | 0.09 |
|  | 3007009 | Hypochondriasis | F452 | 0.08 |
|  | 3001018 | Hysterical reaction | F449 | 0.08 |
|  | 8836934 | Anthropophobia | F401 | 0.07 |
|  | 3008022 | Post-head-injury syndrome | F431 | 0.07 |
|  | 3065005 | Psychogenic voiding dysfunction | F453 | 0.06 |
|  | 8834492 | Social phobia | F401 | 0.06 |
|  | 8835289 | Severe stress reaction | F439 | 0.06 |
|  | 8835105 | Somatoform autonomic dysfunction | F453 | 0.05 |
|  | 3064004 | Gastrointestinal neurosis | F453 | 0.05 |
|  | 3067006 | Psychogenic dizziness | F458 | 0.05 |
|  | 8834902 | Psychogenic polydipsia | F453 | 0.05 |
|  | 3078009 | Psychogenic pain | F454 | 0.05 |
|  | 8834887 | Psychogenic gastric atony | F453 | 0.04 |
|  | 3065009 | Neurogenic voiding dysfunction | F453 | 0.04 |
|  | 3061025 | Psychogenic fever | F453 | 0.04 |
|  | 3064017 | Psychogenic diarrhea | F453 | 0.04 |
|  | 3061019 | Psychogenic dyspnea | F453 | 0.04 |
|  | 8834891 | Psychogenic hyperventilation | F453 | 0.04 |
|  | 3065016 | Bladder hypersensitivity | F453 | 0.04 |
|  | 3064019 | Psychogenic dysphagia | F458 | 0.04 |
|  | 8834542 | Body dysmorphic disorder | F452 | 0.03 |
|  | 8839428 | Agoraphobia | F400 | 0.03 |
|  | 8843724 | Dental phobia | F402 | 0.03 |
|  | 3065007 | Psychogenic constipation | F453 | 0.03 |
|  | 3070002 | Habitual hiccup | F453 | 0.03 |
|  | 3062008 | Psychogenic tachycardia | F453 | 0.02 |
|  | 3008006 | Writer's cramp | F488 | 0.02 |
|  | 8835219 | Persistent somatoform pain disorder | F454 | 0.02 |
|  | 3004016 | Prolonged depressive reaction | F432 | 0.02 |
|  | 8839905 | Undifferentiated somatoform disorder | F451 | 0.02 |
|  | 3068002 | Bruxism | F458 | 0.02 |
|  | 8839600 | Anxiety hysteria | F418 | 0.02 |
|  | 8831074 | Dissociative amnesia | F440 | 0.02 |
|  | 8841368 | Psychogenic tremor | F444 | 0.02 |
|  | 8834895 | Psychogenic flatulence | F453 | 0.02 |
|  | 8831070 | Dissociative motor disorder | F444 | 0.02 |
|  | 3008015 | Chronic psychogenic reaction | F488 | 0.02 |
|  | 8837086 | Specific phobia | F402 | 0.02 |
|  | 3062006 | Psychogenic palpitation | F453 | 0.02 |
|  | 3008017 | Infantile neurosis | F488 | 0.02 |
|  | 8847726 | Globus hystericus | F458 | 0.02 |
|  | 3064002 | Gastric neurosis | F453 | 0.02 |
|  | 8834897 | Psychogenic dyspepsia | F453 | 0.02 |
|  | 8834851 | Esophageal neurosis | F453 | 0.02 |
|  | 3003002 | Compulsive acts | F421 | 0.02 |
|  | 8834896 | Psychogenic syncope | F488 | 0.02 |
|  | 8840962 | Depersonalization disorder | F481 | 0.01 |
|  | 2988012 | Psychogenic seizure | F445 | 0.01 |
|  | 3061020 | Psychogenic aphonia | F444 | 0.01 |
|  | 8842186 | Hypochondriacal disorder | F452 | 0.01 |
|  | 3062009 | Psychogenic arrhythmia | F453 | 0.01 |
|  | 8839939 | Claustrophobia | F402 | 0.01 |
|  |  | *Other codes (k = 96)* |  | *Each <0.01* |
|  |  |  |  |  |
| **Mood disorders** | 2961003 | Depression | F329 | 40.93 |
| (F30–F39) | 3004001 | Depressive state | F329 | 35.72 |
| n = 459,517 | 8842517 | Bipolar affective disorder | F319 | 5.6 |
|  | 3004022 | Depressive neurosis | F341 | 4.18 |
|  | 2966002 | Manic-depressive illness | F319 | 3.52 |
|  | 3004009 | Neurotic depressive state | F341 | 2.01 |
|  | 2960008 | Manic state | F309 | 1.40 |
|  | 8832251 | Dysthymia | F341 | 1.17 |
|  | 2961017 | Adolescent depression | F328 | 0.79 |
|  | 8832914 | Mild depressive episode | F320 | 0.65 |
|  | 8850673 | Bipolar disorder | F319 | 0.63 |
|  | 8835217 | Persistent mood disorder | F349 | 0.52 |
|  | 8837626 | Moderate depressive episode | F321 | 0.32 |
|  | 8849647 | Bipolar II disorder | F318 | 0.30 |
|  | 2969001 | Mood disorder, unspecified | F39 | 0.19 |
|  | 2980001 | Reactive depression | F329 | 0.19 |
|  | 8832941 | Hypomania | F300 | 0.16 |
|  | 8836651 | Bipolar disorder, hypomanic episode | F310 | 0.16 |
|  | 8836650 | Bipolar disorder, mild depressive episode | F313 | 0.15 |
|  | 8839036 | Recurrent mood disorder | F381 | 0.15 |
|  | 8850416 | Premenstrual dysphoric disorder | F328 | 0.15 |
|  | 8832250 | Cyclothymia | F340 | 0.14 |
|  | 2961027 | Atypical depression | F328 | 0.12 |
|  | 8835867 | Severe depressive episode with psychotic symptoms | F323 | 0.12 |
|  | 8836657 | Bipolar disorder, moderate depressive episode | F313 | 0.12 |
|  | 2981006 | Reactive excitement | F308 | 0.08 |
|  | 8836652 | Bipolar disorder, mixed episode | F316 | 0.07 |
|  | 8836653 | Bipolar disorder, manic episode with psychotic symptoms | F312 | 0.06 |
|  | 8835868 | Severe depressive episode without psychotic symptoms | F322 | 0.05 |
|  | 8836655 | Bipolar disorder, manic episode without psychotic symptoms | F311 | 0.05 |
|  | 8835861 | Mania with psychotic symptoms | F302 | 0.04 |
|  | 8836656 | Bipolar disorder, severe depressive episode with psychotic symptoms | F315 | 0.04 |
|  | 2961020 | Hypochondriacal depression | F328 | 0.03 |
|  | 2961026 | Recurrent depression | F339 | 0.03 |
|  | 2960012 | Manic episode | F308 | 0.02 |
|  | 2961005 | Masked depression | F328 | 0.02 |
|  | 3013003 | Aggressive mood disorder | F388 | 0.02 |
|  | 8835862 | Mania without psychotic symptoms | F301 | 0.02 |
|  | 8839043 | Recurrent manic episode | F318 | 0.02 |
|  | 2958003 | Cyclical psychosis | F319 | 0.01 |
|  | 2960005 | Unipolar mania | F309 | 0.01 |
|  | 2961025 | Endogenous depression | F332 | 0.01 |
|  | 2968001 | Post-traumatic depression | F348 | 0.01 |
|  | 8836654 | Bipolar disorder, severe depressive episode without psychotic symptoms | F314 | 0.01 |
|  | 8845740 | Depressive personality disorder | F341 | 0.01 |
|  | 8850488 | Bipolar I disorder | F319 | 0.01 |
|  |  | *Other codes (k = 16)* |  | *Each <0.01* |
|  |  |  |  |  |
| **Eating disorders** | 7833001 | Eating disorder | F509 | 67.82 |
| (F50) | 8834950 | Anorexia nervosa | F500 | 19.62 |
| n = 53,075 | 8834947 | Neurogenic vomiting | F505 | 4.66 |
|  | 3075011 | Anorexia | F508 | 4.47 |
|  | 8830450 | Pica | F508 | 1.16 |
|  | 8834952 | Bulimia nervosa | F502 | 0.92 |
|  | 3075005 | Psychogenic overeating | F504 | 0.57 |
|  | 8839321 | Atypical anorexia nervosa | F501 | 0.48 |
|  | 3075010 | Neurogenic anorexia | F508 | 0.28 |
|  | 8839320 | Atypical bulimia nervosa | F503 | 0.03 |
|  |  |  |  |  |
| **ADHD** | 3073008 | Attention-deficit/hyperactivity disorder | F900 | 94.18 |
| (F90, F98.8) | 8836993 | Hyperkinetic disorder | F909 | 4.84 |
| n = 612,598 | 8836994 | Attention deficit disorder without hyperactivity | F988 | 0.93 |
|  | 8845700 | Hyperkinetic conduct disorder | F901 | 0.06 |
|  |  |  |  |  |
| **ASD** | 8848560 | Autism spectrum disorder | F849 | 79.36 |
| (F84) | 8833660 | Pervasive developmental disorder | F849 | 8.52 |
| n = 872,260 | 8835238 | Autism | F840 | 7.85 |
|  | 2991005 | Asperger syndrome | F845 | 1.30 |
|  | 2990004 | Childhood autism | F840 | 1.14 |
|  | 8844178 | High-functioning autism | F840 | 0.78 |
|  | 8839322 | Atypical autism | F841 | 0.37 |
|  | 2991006 | Autistic intellectual disability | F848 | 0.35 |
|  | 8845311 | Intellectual disability with autistic features | F841 | 0.27 |
|  | 2999001 | Childhood psychosis | F840 | 0.02 |
|  | 2999004 | Childhood psychosis | F840 | 0.01 |
|  | 8831404 | Kanner syndrome | F840 | 0.01 |
|  | 8835239 | Autistic psychopathy | F845 | 0.01 |
|  |  | *Other codes (k = 5)* |  | *Each <0.01* |
|  |  |  |  |  |
| Disease codes refer to the Japanese Standard Disease Name Master codes used in the NDB claims database. | | | | |
| Proportions represent the percentage of each code within its respective diagnostic category. | | |  |  |
| ICD-10 codes were assigned based on the official Japanese Standard Disease Name Master (Version 5.17). | | | | |
| Codes with proportions <0.01% (including those with fewer than 10 cases) are grouped as 'Other codes'. | | |  |  |

**Supplementary Table S2. Baseline incidence rates and estimated changes in new diagnostic trends for mental disorders across pandemic phases: Overall and subgroup-specific interrupted time-series analysis.**

| **Mental Disorder** | **Level-change 1 (School Closure)** | ***unadjusted***  ***p-value*** | ***q-value*** | **Slope-change 1 (Restriction)** | ***unadjusted***  ***p-value*** | ***q-value*** | **Level-change 2 (Lifting)** | ***unadjusted p-value*** | ***q-value*** | **Slope-change 2**  **(Post-Restriction)** | ***unadjusted p-value*** | ***q-value*** |
| --- | --- | --- | --- | --- | --- | --- | --- | --- | --- | --- | --- | --- |
| **Stress-Related Disorders** | |  |  |  |  |  |  |  |  |  |  |  |
| All | 0.953 (0.829, 1.096) | 0.502 | 0.649 | 1.007 (1.000, 1.014) | 0.053 | 0.133 | 0.906 (0.805, 1.020) | 0.102 | 0.207 | 0.988 (0.975, 1.002) | 0.095 | 0.199 |
| Sex |  |  |  |  |  |  |  |  |  |  |  |  |
| Male | 0.905 (0.812, 1.009) | 0.073 | 0.161 | 1.006 (1.000, 1.011) | 0.042 | 0.120 | 0.958 (0.869, 1.056) | 0.385 | 0.536 | 0.987 (0.976, 0.999) | 0.032 | 0.108 |
| Female | 0.995 (0.840, 1.180) | 0.958 | 0.973 | 1.008 (1.000, 1.017) | 0.064 | 0.149 | 0.863 (0.751, 0.993) | 0.040 | 0.120 | 0.989 (0.973, 1.005) | 0.186 | 0.322 |
| Age |  |  |  |  |  |  |  |  |  |  |  |  |
| Age 5–9 | 0.880 (0.803, 0.965) | 0.007 | 0.036* | 1.003 (0.999, 1.008) | 0.145 | 0.261 | 1.017 (0.942, 1.099) | 0.659 | 0.781 | 0.991 (0.981, 1.001) | 0.067 | 0.153 |
| Age 10–14 | 0.935 (0.803, 1.088) | 0.384 | 0.536 | 1.006 (0.999, 1.014) | 0.113 | 0.216 | 0.872 (0.765, 0.994) | 0.040 | 0.120 | 0.992 (0.976, 1.007) | 0.286 | 0.431 |
| Age 15–<19 | 1.017 (0.861, 1.200) | 0.846 | 0.888 | 1.010 (1.001, 1.018) | 0.023 | 0.089 | 0.875 (0.761, 1.007) | 0.062 | 0.149 | 0.984 (0.968, 0.999) | 0.042 | 0.120 |
|  |  |  |  |  |  |  |  |  |  |  |  |  |
| **Mood Disorders** | |  |  |  |  |  |  |  |  |  |  |  |
| All | 1.053 (0.868, 1.278) | 0.601 | 0.735 | 1.005 (0.996, 1.015) | 0.275 | 0.424 | 0.861 (0.737, 1.006) | 0.059 | 0.145 | 0.982 (0.964, 1.000) | 0.048 | 0.128 |
| Sex |  |  |  |  |  |  |  |  |  |  |  |  |
| Male | 0.951 (0.813, 1.112) | 0.528 | 0.669 | 1.005 (0.997, 1.013) | 0.243 | 0.389 | 0.910 (0.802, 1.032) | 0.141 | 0.258 | 0.983 (0.967, 0.999) | 0.033 | 0.108 |
| Female | 1.129 (0.903, 1.411) | 0.286 | 0.431 | 1.006 (0.995, 1.017) | 0.308 | 0.458 | 0.830 (0.693, 0.994) | 0.043 | 0.120 | 0.981 (0.962, 1.001) | 0.064 | 0.149 |
| Age |  |  |  |  |  |  |  |  |  |  |  |  |
| Age 5–9 | 0.878 (0.770, 1.001) | 0.052 | 0.133 | 0.996 (0.990, 1.003) | 0.321 | 0.472 | 0.987 (0.881, 1.106) | 0.825 | 0.885 | 0.985 (0.974, 0.996) | 0.006 | 0.033* |
| Age 10–14 | 1.024 (0.860, 1.219) | 0.787 | 0.868 | 1.003 (0.994, 1.012) | 0.525 | 0.669 | 0.887 (0.772, 1.020) | 0.092 | 0.196 | 0.976 (0.961, 0.992) | 0.003 | 0.017* |
| Age 15–<19 | 1.088 (0.881, 1.345) | 0.433 | 0.577 | 1.007 (0.997, 1.018) | 0.181 | 0.317 | 0.840 (0.709, 0.996) | 0.045 | 0.123 | 0.983 (0.963, 1.003) | 0.088 | 0.191 |
|  |  |  |  |  |  |  |  |  |  |  |  |  |
| **Eating disorders** | | | |  |  |  |  |  |  |  |  |  |
| All | 1.090 (0.910, 1.307) | 0.349 | 0.508 | 1.004 (0.995, 1.012) | 0.397 | 0.546 | 1.064 (0.912, 1.241) | 0.429 | 0.577 | 0.986 (0.969, 1.003) | 0.104 | 0.208 |
| Sex |  |  |  |  |  |  |  |  |  |  |  |  |
| Male | 0.712 (0.619, 0.819) | <0.001 | <0.001* | 1.012 (1.004, 1.020) | 0.003 | 0.017* | 1.214 (1.076, 1.370) | 0.002 | 0.013* | 0.981 (0.965, 0.997) | 0.017 | 0.073 |
| Female | 1.284 (1.045, 1.576) | 0.017 | 0.073 | 1.000 (0.991, 1.010) | 0.948 | 0.973 | 1.000 (0.834, 1.199) | 0.997 | 0.997 | 0.989 (0.970, 1.008) | 0.241 | 0.389 |
| Age |  |  |  |  |  |  |  |  |  |  |  |  |
| Age 5–9 | 0.709 (0.612, 0.822) | <0.001 | <0.001* | 1.016 (1.007, 1.024) | <0.001 | <0.001* | 1.112 (0.948, 1.305) | 0.191 | 0.322 | 0.982 (0.966, 0.997) | 0.022 | 0.088 |
| Age 10–14 | 1.028 (0.878, 1.204) | 0.728 | 0.832 | 1.003 (0.996, 1.010) | 0.429 | 0.577 | 1.057 (0.938, 1.192) | 0.362 | 0.521 | 0.983 (0.967, 0.998) | 0.025 | 0.091 |
| Age 15–<19 | 1.316 (1.051, 1.648) | 0.017 | 0.073 | 1.000 (0.989, 1.010) | 0.954 | 0.973 | 1.013 (0.815, 1.260) | 0.905 | 0.942 | 0.993 (0.970, 1.016) | 0.552 | 0.693 |
|  |  |  |  |  |  |  |  |  |  |  |  |  |
| **ADHD** |  |  |  |  |  |  |  |  |  |  |  |  |
| All | 0.919 (0.847, 0.997) | 0.043 | 0.120 | 1.004 (0.999, 1.008) | 0.106 | 0.209 | 0.994 (0.938, 1.053) | 0.833 | 0.885 | 0.982 (0.974, 0.990) | <0.001 | <0.001* |
| Sex |  |  |  |  |  |  |  |  |  |  |  |  |
| Male | 0.913 (0.840, 0.992) | 0.032 | 0.108 | 1.003 (0.998, 1.008) | 0.191 | 0.322 | 1.010 (0.952, 1.071) | 0.751 | 0.843 | 0.980 (0.972, 0.988) | <0.001 | <0.001* |
| Female | 0.940 (0.865, 1.021) | 0.141 | 0.258 | 1.005 (1.001, 1.009) | 0.024 | 0.090 | 0.957 (0.902, 1.016) | 0.151 | 0.268 | 0.984 (0.976, 0.992) | <0.001 | <0.001* |
| Age |  |  |  |  |  |  |  |  |  |  |  |  |
| Age 0–4 | 0.998 (0.900, 1.107) | 0.970 | 0.978 | 1.001 (0.995, 1.006) | 0.837 | 0.885 | 1.019 (0.912, 1.138) | 0.742 | 0.840 | 0.972 (0.959, 0.985) | <0.001 | <0.001* |
| Age 5–9 | 0.921 (0.848, 1.000) | 0.051 | 0.133 | 1.002 (0.997, 1.007) | 0.382 | 0.536 | 1.022 (0.960, 1.088) | 0.498 | 0.649 | 0.978 (0.970, 0.987) | <0.001 | <0.001* |
| Age 10–14 | 0.879 (0.819, 0.944) | <0.001 | <0.001* | 1.003 (0.999, 1.007) | 0.133 | 0.250 | 0.987 (0.937, 1.040) | 0.622 | 0.744 | 0.985 (0.977, 0.992) | <0.001 | <0.001* |
| Age 15–<19 | 0.969 (0.860, 1.091) | 0.603 | 0.735 | 1.012 (1.006, 1.018) | <0.001 | <0.001* | 0.955 (0.880, 1.037) | 0.274 | 0.424 | 0.983 (0.971, 0.995) | 0.007 | 0.036* |
|  |  |  |  |  |  |  |  |  |  |  |  |  |
| **ASD** |  |  |  |  |  |  |  |  |  |  |  |  |
| All | 0.959 (0.899, 1.024) | 0.215 | 0.353 | 1.004 (1.000, 1.007) | 0.037 | 0.118 | 1.014 (0.961, 1.069) | 0.615 | 0.743 | 0.981 (0.973, 0.988) | <0.001 | <0.001* |
| Sex |  |  |  |  |  |  |  |  |  |  |  |  |
| Male | 0.948 (0.894, 1.004) | 0.069 | 0.155 | 1.003 (1.000, 1.006) | 0.097 | 0.200 | 1.030 (0.979, 1.084) | 0.254 | 0.401 | 0.981 (0.974, 0.988) | <0.001 | <0.001* |
| Female | 0.989 (0.907, 1.078) | 0.803 | 0.878 | 1.006 (1.001, 1.010) | 0.011 | 0.052 | 0.978 (0.917, 1.043) | 0.499 | 0.649 | 0.980 (0.970, 0.989) | <0.001 | <0.001* |
| Age |  |  |  |  |  |  |  |  |  |  |  |  |
| Age 0–4 | 0.982 (0.900, 1.070) | 0.673 | 0.790 | 0.999 (0.995, 1.004) | 0.830 | 0.885 | 0.989 (0.916, 1.067) | 0.767 | 0.854 | 0.977 (0.968, 0.985) | <0.001 | <0.001* |
| Age 5–9 | 0.933 (0.885, 0.983) | 0.009 | 0.044* | 1.004 (1.001, 1.007) | 0.018 | 0.074 | 1.038 (0.992, 1.086) | 0.111 | 0.215 | 0.983 (0.976, 0.989) | <0.001 | <0.001* |
| Age 10–14 | 0.956 (0.892, 1.025) | 0.204 | 0.339 | 1.004 (1.000, 1.008) | 0.033 | 0.108 | 1.011 (0.953, 1.074) | 0.708 | 0.823 | 0.982 (0.973, 0.990) | <0.001 | <0.001* |
| Age 15–<19 | 1.021 (0.915, 1.139) | 0.714 | 0.823 | 1.011 (1.005, 1.016) | <0.001 | <0.001* | 1.022 (0.948, 1.103) | 0.570 | 0.708 | 0.975 (0.963, 0.987) | <0.001 | <0.001* |

Values are expressed as estimates (95% confidence intervals).
Level/Slope-changes represent estimated immediate shift and change in trend, respectively, at each transition point.
q < 0.05* after FDR correction (Benjamini–Hochberg method).
Abbreviations: ADHD, attention-deficit/hyperactivity disorder; ASD, autism spectrum disorder.
